# Supplementary material for: Current applications and outcomes of AI-driven adaptive learning systems in physical rehabilitation science education: A scoping review protocol
Source: PLoS One. 2025 Jun 11;20(6):e0325649. doi: 10.1371/journal.pone.0325649 (PMC12157232; doi:10.1371/journal.pone.0325649)
Supplement: S2 Appendix — (DOCX) [file pone.0325649.s002.docx]

**S2 Appendix: PubMed search strategy**

Search: (("Artificial Intelligence" OR AI OR "Machine Learning" OR "Neural Networks" OR Algorithms OR "Deep Learning" OR "Generative AI" OR "Natural Language Processing" OR LLM OR "Large Language Model" OR "Data Mining") AND (Learning OR "Adaptive Learning" OR Education OR "Computer-Assisted Instruction" OR "Expert System*" OR Computer-Assisted OR "Personalized learning" OR "personalised learning" OR "Educational measurement." OR "Problem-Based Learning" OR "intelligent tutoring system*")) AND (Rehabilitation OR "Occupational Therap*" OR Orthotics OR Prosthetic OR Chiropract* OR "Recreation Therap*" OR "Rehabilitation counselling" OR "Sport therap*" OR Kinesiology OR Audiology OR "Speech-Language Pathology" OR "speech Language Patholog*" or "Speech Disorders" OR "Vocational therap*" OR physiotherap* OR "physical therap*" OR "manual therapy" OR "exercise therapy") Filters: from 2019 - 2024 Sort by: Most Recent

(("Artificial Intelligence"[All Fields] OR ("antagonists and inhibitors"[MeSH Subheading] OR ("antagonists"[All Fields] AND "inhibitors"[All Fields]) OR "antagonists and inhibitors"[All Fields] OR "ai"[All Fields]) OR "Machine Learning"[All Fields] OR "Neural Networks"[All Fields] OR ("algorithm s"[All Fields] OR "algorithmic"[All Fields] OR "algorithmically"[All Fields] OR "algorithmics"[All Fields] OR "algorithmization"[All Fields] OR "algorithms"[MeSH Terms] OR "algorithms"[All Fields] OR "algorithm"[All Fields]) OR "Deep Learning"[All Fields] OR "Generative AI"[All Fields] OR "Natural Language Processing"[All Fields] OR "LLM"[All Fields] OR "Large Language Model"[All Fields] OR "Data Mining"[All Fields]) AND ("learning"[MeSH Terms] OR "learning"[All Fields] OR "learn"[All Fields] OR "learned"[All Fields] OR "learning s"[All Fields] OR "learnings"[All Fields] OR "learns"[All Fields] OR "Adaptive Learning"[All Fields] OR ("educability"[All Fields] OR "educable"[All Fields] OR "educates"[All Fields] OR "education"[MeSH Subheading] OR "education"[All Fields] OR "educational status"[MeSH Terms] OR ("educational"[All Fields] AND "status"[All Fields]) OR "educational status"[All Fields] OR "education"[MeSH Terms] OR "education s"[All Fields] OR "educational"[All Fields] OR "educative"[All Fields] OR "educator"[All Fields] OR "educator s"[All Fields] OR "educators"[All Fields] OR "teaching"[MeSH Terms] OR "teaching"[All Fields] OR "educate"[All Fields] OR "educated"[All Fields] OR "educating"[All Fields] OR "educations"[All Fields]) OR "Computer-Assisted Instruction"[All Fields] OR "expert system*"[All Fields] OR "Computer-Assisted"[All Fields] OR "Personalized learning"[All Fields] OR "personalised learning"[All Fields] OR "educational measurement"[All Fields] OR "Problem-Based Learning"[All Fields] OR "intelligent tutoring system*"[All Fields]) AND ("rehabilitant"[All Fields] OR "rehabilitants"[All Fields] OR "rehabilitate"[All Fields] OR "rehabilitated"[All Fields] OR "rehabilitates"[All Fields] OR "rehabilitating"[All Fields] OR "rehabilitation"[MeSH Terms] OR "rehabilitation"[All Fields] OR "rehabilitations"[All Fields] OR "rehabilitative"[All Fields] OR "rehabilitation"[MeSH Subheading] OR "rehabilitation s"[All Fields] OR "rehabilitational"[All Fields] OR "rehabilitator"[All Fields] OR "rehabilitators"[All Fields] OR "occupational therap*"[All Fields] OR ("orthotic devices"[MeSH Terms] OR ("orthotic"[All Fields] AND "devices"[All Fields]) OR "orthotic devices"[All Fields] OR "orthotics"[All Fields] OR "orthotic"[All Fields]) OR ("prosthetic"[All Fields] OR "prosthetically"[All Fields] OR "prosthetics"[All Fields]) OR "chiropract*"[All Fields] OR "recreation therap*"[All Fields] OR "Rehabilitation counselling"[All Fields] OR "sport therap*"[All Fields] OR ("kinesiology zagreb"[Journal] OR "kinesiology"[All Fields]) OR ("audiology"[MeSH Terms] OR "audiology"[All Fields]) OR "Speech-Language Pathology"[All Fields] OR "speech language patholog*"[All Fields] OR "Speech Disorders"[All Fields] OR "vocational therap*"[All Fields] OR "physiotherap*"[All Fields] OR "physical therap*"[All Fields] OR "manual therapy"[All Fields] OR "exercise therapy"[All Fields])) AND (2019:2024[pdat])

**Translations**

AI: "antagonists and inhibitors"[Subheading] OR ("antagonists"[All Fields] AND "inhibitors"[All Fields]) OR "antagonists and inhibitors"[All Fields] OR "ai"[All Fields]

Algorithms: "algorithm's"[All Fields] OR "algorithmic"[All Fields] OR "algorithmically"[All Fields] OR "algorithmics"[All Fields] OR "algorithmization"[All Fields] OR "algorithms"[MeSH Terms] OR "algorithms"[All Fields] OR "algorithm"[All Fields]

Learning: "learning"[MeSH Terms] OR "learning"[All Fields] OR "learn"[All Fields] OR "learned"[All Fields] OR "learning's"[All Fields] OR "learnings"[All Fields] OR "learns"[All Fields]

Education: "educability"[All Fields] OR "educable"[All Fields] OR "educates"[All Fields] OR "education"[Subheading] OR "education"[All Fields] OR "educational status"[MeSH Terms] OR ("educational"[All Fields] AND "status"[All Fields]) OR "educational status"[All Fields] OR "education"[MeSH Terms] OR "education's"[All Fields] OR "educational"[All Fields] OR "educative"[All Fields] OR "educator"[All Fields] OR "educator's"[All Fields] OR "educators"[All Fields] OR "teaching"[MeSH Terms] OR "teaching"[All Fields] OR "educate"[All Fields] OR "educated"[All Fields] OR "educating"[All Fields] OR "educations"[All Fields]

Rehabilitation: "rehabilitant"[All Fields] OR "rehabilitant's"[All Fields] OR "rehabilitants"[All Fields] OR "rehabilitate"[All Fields] OR "rehabilitated"[All Fields] OR "rehabilitates"[All Fields] OR "rehabilitating"[All Fields] OR "rehabilitation"[MeSH Terms] OR "rehabilitation"[All Fields] OR "rehabilitations"[All Fields] OR "rehabilitative"[All Fields] OR "rehabilitation"[Subheading] OR "rehabilitation's"[All Fields] OR "rehabilitational"[All Fields] OR "rehabilitator"[All Fields] OR "rehabilitators"[All Fields]

Orthotics: "orthotic devices"[MeSH Terms] OR ("orthotic"[All Fields] AND "devices"[All Fields]) OR "orthotic devices"[All Fields] OR "orthotics"[All Fields] OR "orthotic"[All Fields]

Prosthetic: "prosthetic"[All Fields] OR "prosthetically"[All Fields] OR "prosthetics"[All Fields]

Audiology: "audiology"[MeSH Terms] OR "audiology"[All Fields]
