# Supplementary material for: Current applications and outcomes of AI-driven adaptive learning systems in physical rehabilitation science education: A scoping review protocol
Source: PLoS One. 2025 Jun 11;20(6):e0325649. doi: 10.1371/journal.pone.0325649 (PMC12157232; doi:10.1371/journal.pone.0325649)
Supplement: Attachment — Submitted filename: Response to Reviewers.pdf [file pone.0325649.s004.pdf]

Oyindolapo Komolafe
Faculty of Health Sciences,
Western University,
London, Ontario, Canada
okomola@uwo.ca

April 30, 2025

Editorial TeamS
PLOS ONE

Subject: Response to Reviewers

Dear Editorial Team,

The following are the responses to the questions raised by the reviewers:

Question: Please let the readers know why your search didn't include optometry and audiology.

Audiology has been included in the search strategy. The definition of Physical Rehabilitation is stated to justify the inclusion of the disciplines (lines 22-28)

Question: You have focused on "Physical rehabilitation". Please let the readers know why you include cognitive therapy.

Cognitive therapy has been removed from the protocol and search strategy

Question: Although "adaptive learning" has been considered in the process, the main questions do not reflect it

As outlined in the introduction (lines 1-8), we define adaptive learning as the use of AI-driven technology to personalize the learning experience, including aspects such as content delivery, evaluation, and assessment. Our intention with the current formulation of the research questions is to take a broad and inclusive approach to understanding how AI technologies—particularly those with adaptive capabilities—are being utilized within physical rehabilitation science education. - While the term adaptive learning is not explicitly mentioned in the research questions, it is conceptually embedded in our first question, which explores the types of AI models and their applications in enhancing teaching and learning. This phrasing was chosen to capture both general

AI applications and those that include adaptive learning features such as personalization, real-time feedback, and learner-specific modifications.

Question: Please define education clearly; does it also include evaluation and assessment (It should be).

Thank you for this thoughtful observation. We agree that education, particularly in the context of adaptive learning, should be clearly defined to encompass not only instructional delivery but also evaluation and assessment. To address this, we have revised the introduction to explicitly define education as a multidimensional process that includes teaching, learning, assessment, and feedback. This definition aligns with the mechanisms of adaptive learning systems, which rely heavily on continuous assessment to personalize learning experiences.

Sincerely,
Oyindolapo Komolafe
Western University
