# Supplementary material for: Current applications and outcomes of AI-driven adaptive learning systems in physical rehabilitation science education: A scoping review protocol
Source: PLoS One. 2025 Jun 11;20(6):e0325649. doi: 10.1371/journal.pone.0325649 (PMC12157232; doi:10.1371/journal.pone.0325649)
Supplement: Attachment — Submitted filename: Response to Reviewers.docx [file pone.0325649.s003.docx]

Oyindolapo Komolafe
Faculty of Health Sciences,

Western University,

London, Ontario, Canada
[okomola@uwo.ca](mailto:okomola@uwo.ca)

February 24, 2025

Editorial TeamS
PLOS ONE

**Subject:** Response to Reviewers

Dear Editorial Team,

Thank you for the editorial comments. The latex template on plos one website is used for formatting the protocol. The following are the responses to the questions raised by the reviewers:

## **Question: Please consider PubMed, Scopus, Pedro and other specific databases in rehabilitation sciences as additional databases. Medline is subcategory of PubMed; you can delete it & considering that many journals are now indexed in the Scopus database (under Elsevier), I strongly recommend including Scopus as part of the systematic search strategy.**

The search database is updated to include Scopus. .The databases searched are Scopus, PubMed, CINAHL, Education Resources Information Center (ERIC), Association for Computing Machinery (ACM), ProQuest Education Journal, Web of Science, ProQuest Dissertations & Theses Global, and IEEE Digital Library

## **Question: Why is this project mix method?**

## The scoping review is not a mixed method, thus removed from the abstract

## **Question: The type of review questions is different in various study design. If there are enough literatures for conducting the present review study, please limit this research project to one of study design category for example observational studies.**

This research is a scoping review which typically asks broad questions about the scope of research on a topic. According to Peter et al (2020), a scoping review is used to map concepts, identify and analyse gaps, and inform future research. As such, inclusion of different study designs is encouraged in scoping reviews.

## **Question: Please rewrite the background (the abstract) in 3 short sentences: the importance of AI in rehabilitation education, research gap, and the primary outcome**

The introduction section of the abstract is reduced to meet the requirements

## **Question: If it is possible, please revise review questions according to one of review questions format.**

We have considered the reviewer’s comment, however, because this research is a scoping review, which typically asks broad questions about the scope of research on a topic, we are confident in the diversity of questions. According to Peter et al (2020), a scoping review is used to map concepts, identify and analyse gaps, and inform future research.

## **Question: Please consider time farmwork and study design as inclusion and exclusion criteria.**

The inclusion criteria for a scoping review is contingent on the questions posed. The PCC is stipulated (Population, Concept, and Context). Per Peter (2020) we are attempting to develop a broad understanding of the current state of literature pertaining to our research question. As AI tools in education are a relatively new advancement, we do not anticipate a large volume of research especially when limited to only rehabilitation training contexts. Accordingly, we have an opportunity to conduct a review that is both comprehensive and feasible. We respectfully suggest that retaining the broad search strategy is the best approach for our objectives.

**Question: Please revise key word for searching according to EMTREE and MeSH terms**.

The search query for Medline and PubMed with the MeSH terms is added to Appendix. The search strategy combines structure database-specific subject headings and keywords/ synonyms of the following concepts:

- AI models
- Adaptive learning systems
- Rehabilitation science (physical therapy, occupational therapy, orthotics, prosthetics, chiropractic, recreation therapy, sports therapy, rehabilitation counseling, kinesiology, cognitive therapy, speech-language pathology and vocational therapy)

## **Question: Is a quality assessment mandatory in scoping review studies? Please provide clarity on this in the manuscript.** In the discussion section, please elaborate on the rationale for not evaluating the quality of the included studies.

In scoping review, critical appraisal (risk of bias assessment) is not required (Munn et al ,2018 Table 1) but reviewers may decide to access and report bias if it is relevant for their objectives. In our case the goal is to determine the current state of literature, what tools are being used and how, rather than assess the quality or risk of bias of the published manuscripts. This will have the added value of enabling our intention of a broad and comprehensive overview while remaining feasible. Paragraph in this regard has been added to discussion with reference to Munn et al. (lines 200-203)

## **Question: In the methodology section, [Line #], kindly introduce JBI as the abbreviation for the Joanna Briggs Institute upon its first mention.**

We have updated the protocol to address the suggestion

## **Question: Since educational systems vary between countries, it would be helpful to specify the country of each included study and the educational level of participants (e.g., undergraduate or postgraduate students).**

Country where study tool place and participants level of education has been added to the Data Extraction table. Thank you for the helpful comment.

## **Question: The education in rehab sciences (in practice) is a broad and diverse field. In case you find enough study on all the disciplines, discussing the results would be challenging.**

The scoping review is focused on physical rehabilitation science which includes: physical therapy, occupational therapy, orthotics, prosthetics, chiropractic, recreation therapy, sports therapy, rehabilitation counseling, kinesiology, cognitive therapy, speech-language pathology and vocational therapy. We are not including psychological / mental health rehabilitation. Our pilot searches indicate the overall volume of relevant literature will not be so large as to make interpretation and discussion problematic.

## **Question: Please let the reader know if you include general topics and courses like Anatomy.**

At this point we are keeping the scope intentionally broad meaning we will include any peer-reviewed research exploring use of any AI tool in any physical rehabilitation training program, regardless of whether that is for a single relevant course like anatomy, or used across a program (e.g., for interactive case studies or learning evaluations). The limiting factor is that the manuscript must explicitly state that the course was included as part of a physical rehabilitation professional training program, rather than a general undergraduate course, for example.

## **Question: Do you use any AI tool in any part of your study?**

No, there is no AI tool used for the scoping review.

Sincerely,
Oyindolapo Komolafe

Western University

**References**

Munn, Z., Peters, M.D.J., Stern, C. *et al.* Systematic review or scoping review? Guidance for authors when choosing between a systematic or scoping review approach. *BMC Med Res Methodol* **18**, 143 (2018). <https://doi.org/10.1186/s12874-018-0611-x>

Peters MDJ, Godfrey C, McInerney P, Munn Z, Tricco AC, Khalil, H. Scoping Reviews (2020). Aromataris E, Lockwood C, Porritt K, Pilla B, Jordan Z, editors. *JBI Manual for Evidence Synthesis*. JBI; 2024. Available from: [https://synthesismanual.jbi.global](https://synthesismanual.jbi.global/). <https://doi.org/10.46658/JBIMES-24-09>
